# FGF10 and the Mystery of Duodenal Atresia in Humans

**DOI:** 10.3389/fgene.2018.00530

**Published:** 2018-11-09

**Authors:** Warwick J. Teague, Matthew L. M. Jones, Leanne Hawkey, Ian M. Smyth, Angelique Catubig, Sebastian K. King, Gulcan Sarila, Ruili Li, John M. Hutson

**Affiliations:** ^1^F. Douglas Stephens Surgical Research Laboratory, Murdoch Children’s Research Institute, Melbourne, VIC, Australia; ^2^Department of Paediatrics, The University of Melbourne, Melbourne, VIC, Australia; ^3^Discipline of Surgery, Sydney Medical School, The University of Sydney, Sydney, NSW, Australia; ^4^Department of Paediatric Surgery, The Royal Children’s Hospital, Melbourne, VIC, Australia; ^5^Australian Phenomics Network, Department of Anatomy and Developmental Biology, Monash University, Melbourne, VIC, Australia; ^6^Department of Anatomy and Developmental Biology, Monash Biomedicine Discovery Institute, Monash University, Melbourne, VIC, Australia; ^7^Department of Biochemistry and Molecular Biology, Monash Biomedicine Discovery Institute, Monash University, Melbourne, VIC, Australia; ^8^Department of Gastroenterology and Clinical Nutrition, The Royal Children’s Hospital, Melbourne, VIC, Australia; ^9^Department of Urology, The Royal Children’s Hospital, Melbourne, VIC, Australia

**Keywords:** duodenal obstruction, congenital intestinal atresia, fibroblast growth factor 10, morphogenesis, models, animal, CRISPR-Cas systems

## Abstract

**Background:** Duodenal atresia (DA) is a congenital obstruction of the duodenum, which affects 1 in 7000 pregnancies and requires major surgery in the 1st days of life. Three morphological DA types are described. In humans, the association between DA and Down syndrome suggests an underlying, albeit elusive, genetic etiology. In mice, interruption of fibroblast growth factor 10 (*Fgf10*) gene signaling results in DA in 30–50% of embryos, supporting a genetic etiology. This study aims to validate the spectrum of DA in two novel strains of *Fgf10* knock-out mice, in preparation for future and translational research.

**Methods:** Two novel CRISPR *Fgf10* knock-out mouse strains were derived and embryos generated by heterozygous plug-mating. E15.5–E19.5 embryos were genotyped with respect to *Fgf10* and micro-dissected to determine the presence and type of DA.

**Results:** One twenty seven embryos (32 wild-type, 34 heterozygous, 61 null) were analyzed. No wild-type or heterozygous embryos had DA. However, 74% of *Fgf10* null embryos had DA (49% type 1, 18% type 2, and 33% type 3).

**Conclusion:** Our CRISPR-derived strains showed higher penetrance of DA due to single-gene deletion of *Fgf10* in mice than previously reported. Further, the DA type distribution in these mice more closely reiterated that observed in humans. Future experiments will document RNA and protein expression of FGF10 and its key downstream signaling targets in normal and atretic duodenum. This includes exploitation of modern, high-fidelity developmental tools, e.g., *Fgf10*^flox/+^–tomato^flox/flox^ mice.

## Introduction

Duodenal atresia (DA) is an important congenital cause of bowel obstruction in newborns. Often diagnosed prenatally on ultrasound scan or postnatally on abdominal radiograph, it requires intensive care after birth and major surgery in the 1st days of life. Three morphological types of DA are described, reflecting increasing degrees of obstruction and discontinuity; type 1: bowel continuity but luminal obstruction or stenosis, type 2: bowel discontinuity with a connecting “bridge” of tissue, and type 3: bowel discontinuity with complete separation (Skandalakis and Gray, 1994). While modern surgical and neonatal management of DA achieves survival in more than 95% of cases (Khan et al., 2017), the embryological etiology of this condition remains an unanswered question amongst researchers, clinicians and patients’ families.

In 1900, the Viennese anatomist Julius Tandler meticulously studied 11 human embryos with apparently normal gut, and theorized that the duodenum underwent a normal “solid cord” phase during development, secondary to exuberant endodermal (epithelial) growth. Further, he suggested failure of this proposed cord to re-canalize accounted for DA (Tandler, 1900). At the time, Tandler himself cautiously stated, “*It is clear to me that the opinion represented here does not exceed the status of a new hypothesis, and is not meant to exceed this*” (Nichol et al., 2011). Despite this caution, Tandler’s theory was rapidly and widely adopted as dogma, and went largely unchallenged for many years. Merrot et al. (2006) were amongst the first to counter the “recanalization theory,” asserting that it failed to explain the different morphological types and other variability seen in humans with DA. Furthermore, clinical reports have suggested a genetic basis for DA, with an autosomal recessive inheritance pattern reported for some cases (Berant and Kahana, 1970; Gross et al., 1996; Lambrecht and Kluth, 1998), and the well-recognized association with Down syndrome (Trisomy 21), which occurs in approximately one fifth of DA cases (Khan et al., 2017).

Fairbanks et al. (2004) were the first to report a link between DA and fibroblast growth factor (FGF) pathways in mice, specifically fibroblast growth factor 10 (FGF10) and its receptor fibroblast growth factor receptor 2b (FGFR2b). The FGF family of signaling molecules composed of at least 22 members involved in different aspects of organogenesis (Ornitz and Itoh, 2001), of which FGF10 is associated with instructive mesenchymal/epithelial interactions, occurring during budding and branching morphogenesis. The homozygous deletion of the *Fgf10* gene results in mice which are non-viable after birth due to lung agenesis, also exhibiting defects of the limbs, anterior pituitary gland, salivary glands, inner ear, teeth, skin, and skull (De Moerlooze et al., 2000; Mailleux et al., 2002). Since 2004, several reports have highlighted the importance of the FGF10-FGFR2b signaling pathway in DA as well as other congenital gut malformations such as caecal atresia (Kanard et al., 2005; Fairbanks et al., 2006; Nichol et al., 2011; Botham et al., 2012; Reeder et al., 2012).

We report here our experiences with an *Fgf10* knock-out mouse model, the strains for which were developed using novel CRISPR/Cas9 techniques. Our investigations to date have focused upon characterization of these strains, with a view toward hypothesis-driven determination of the genetic etiology of DA. Herein, we also discuss our future directions and investigations, which aim to make optimal use of modern and powerful developmental biology tools.

## Materials and Methods

### Mouse Strain Derivation

Mutant animals were produced via CRISPR/Cas9 injection of C57/Bl6 oocytes by Monash University as a node of the Australian Phenomics Network (APN). In contrast to Yasue et al. (2014), who used CRISPR technique to target exon 1 of the *Fgf10* gene, our strains were derived using RNA guides targeting exon 3, designed with the aim of minimizing “off-target” events.

Thus, two novel murine strains were developed, the first with a 7 bp duplication and 140 bp deletion (B6-*Fgf10*<c.[464_470dup; 506_645del]APNMu>), and the second with a 13 bp deletion (B6-*Fgf10*<c.495_507delAPNMu>) (Eppig et al., 2006). These strains are coined here as “tm1” (464_470dup; 506_645del) and “tm2” (495_507del), respectively.

### Animal Husbandry and Mating Strategies

Mice were maintained in a temperature- (24°C) and lighting- (14:10 h light-dark cycle) controlled room with free access to food and water within the accredited, institutional animal facility, and cared for in strict adherence with animal welfare and ethical requirements.

To establish breeding colonies for each strain, wild-type and heterozygous mice were mated, and liveborn pups genotyped to inform successive matings; see genotyping protocols below. *Fgf10* mutation is fatal in its homozygous (null) form due to lung agenesis (Sekine et al., 1999). Therefore, heterozygous plug-mating was reserved for experimental use only, with the aim of generating null, heterozygous, and wild-type embryos. For these experimental matings, pregnant dams were humanely culled at timed gestations to provide timed embryos ranging from E15.5 to E19.5.

### Mouse Strain Genotype Characterization

Animals were genotyped by PCR analysis of DNA, extracted from tail or ear clippings. For the tm1 strain, a common forward primer was used (5′-GGAGTGTAGATCATTACATGGC-3′) with a tm1-specific reverse primer (5′-GTGAGGATACCATCTCTTTCTGTCC-3′) to produce a wild-type allele of 348 bp and mutant allele of 215 bp. For tm2, the same forward primer was used (5′-GGAGTGTAGATCATTACATGGC-3′) with the tm2-specific reverse primer (5′-GAATTCAGGGCTATGTCTTTGC-3′) producing a wild-type allele of 242 and 148 bp (390 bp before Nsi1 digestion) and mutant allele of 377 bp. The full details of DNA extraction and standard PCR protocols used are provided as Supplementary Material.

### RNA and RT-qPCR Analysis

Total RNA was extracted using the RNeasy mini column (Qiagen, Cat: 74104), treated with DNase I (Qiagen, Cat: 79254), and the concentration was determined using NanoDrop spectrophotometer (Thermo Scientifics, 2000). Complementary DNA (cDNA) was synthesized using Bio-Rad iScript Advanced cDNA kit (Bio-Rad, Cat: 170-8842). Gene expression (2 μl of cDNA) was measured by GoTaq qPCR (Promega, Cat: A6001) for real time quantitative polymerase chain reaction (RT-qPCR). Nucleotide sequences for *Fgf10* mRNA (*Fw* 5′-CACCTATGCATCTTTTAACTGGC-3′) (*Rv* 5′-TCTATGTTTGGATCGTCATGGGG-3′) was used and expression was normalized to *Rpl32* (*Fw* 5′-GAGGACCAAGAAGTTCATCAGG-3′) (*Rv* 5′-CATTGTGGACCAGGAACTTGC-3′). The results were analyzed using the 7500 SDS software and relative expression was calculated to determine the fold change. Statistical analysis was performed in Prism 7.0 (GraphPad Software). Error bars on the results are presented as a standard error of the mean (SEM), and a *p*-value of <0.05 was considered significant. Statistical significance was evaluated using Student’s unpaired *t*-test. The full details of RT-qPCR protocols used are provided as Supplementary Material.

### Mouse Strain Phenotype Characterization

Embryos of gestation E15.5–E19.5 were humanely collected, culled and micro-dissected, during which key phenotypic features were recorded using bright-field microscopy. Greatest attention was shown to the morphology of the foregut, and determination made as to the presence and type of DA where applicable. Where ambiguous, duodenal morphology, and patency was further investigated using either wholemount immunostaining or paint-filling as adapted from techniques to delineate embryonic murine inner ear morphology (Morsli et al., 1998). The entirety of the duodenum from the pylorus to the duodenojejunal flexure was preserved intact, with back-light microscopy to confirm patency. Duodenal type was determined by consensus of two investigators at the time of dissection, and then representative images secondarily re-assessed independently by two or more authors blinded to the original type determination to provide internal validation.

### Ethical Considerations

Ethics approval for the experiment was granted from the Murdoch Children’s Research Institute (MCRI) Animal Ethics Committee (#A792), and the MCRI Institutional Biosafety Committee (#215-2014 PC1 NLRD).

## Results

### Population

A total of 280 embryos were collected for this study (144 tm1 and 136 tm2 embryos).

Of the 144 tm1 strain embryos, genotyping determined 42 (29.2%) embryos to be wild-type, 74 (51.4%) heterozygous, and 28 (19.4%) *Fgf10* null embryos. With respect to gestation, 70 embryos were collected at E15.5, 32 at E16.5, 23 at E17.5, and 19 at E18.5. In total, 67 tm1 embryos were further micro-dissected, that being 20 wild-type, 19 heterozygous, and all 28 *Fgf10* null embryos.

Of the tm2 strain embryos, genotyping determined 35 (25.7%) embryos to be wild-type, 68 (50%) heterozygous, and 33 (24.3%) *Fgf10* null embryos. Eighteen embryos were collected at E15.5, 29 at E16.5, 25 at E17.5, 48 at E18.5, and 16 at E19.5. In total, 60 tm2 strain embryos were micro-dissected, that being 12 wild-type, 15 heterozygous, and all 33 *Fgf10* null embryos.

### Genotype Characterization

**FIGURE 1:**
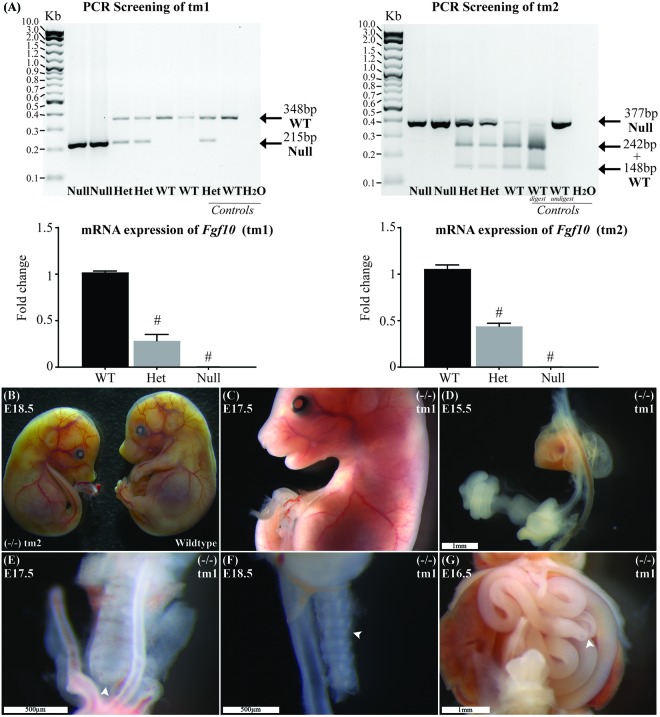
Genotype and general phenotype characterization. **(A)** Product from standard and RT-qPCR shows genotyping and RNA expression for both tm1 and tm2 strains. **(B)** General phenotype of a representative *Fgf10* null embryo (left embryo) with the expected lack of limbs and smaller than body-size when compared with wild type litter mate (right embryo). Fgf10 null embryos also demonstrated: abnormal facies **(C)**, lung agenesis **(D–F)**, anomalous tracheal rings **(F)**, and caecal atresia **(G)**. Arrows highlight features of interest. Gestational ages for panels **B–G** are as stated. ^#^*p*-value < 0.001, comparing either *Fgf10* heterozygous or null mice to their wild-type littermates using Student’s *t*-test.

The product from classic PCR genotyping confirmed expected genetics according to the tm1 and tm2 mutations (Figure 1A). The PCR product for each strain was formally sequenced to confirm correct action of the primers in each protocol. Semiquantitative PCR showed RNA expression for heterozygote and *Fgf10* null embryos in both tm1 and tm2 strains (Figure 1A). With wild-type litter mates providing controls for *Fgf10* gene expression in both strains, the heterozygous embryos expressed approximately half the *Fgf10* (tm1 = 42.29% and tm2 = 51.16%), and no expression was seen in *Fgf10* null embryos.

### General Phenotype Characterization

The general phenotype of both tm1 and tm2 strain *Fgf10* null embryos was consistent with previously reported *Fgf10* knock-out mouse strains (Sekine et al., 1999; Sala et al., 2011; Teshima et al., 2016). These *Fgf10* null embryos demonstrated characteristic lack of limbs and smaller than normal body size (Figures 1B,C), abnormal facies (Figure 1C), lung agenesis (Figures 1D–F) anomalous tracheal rings (Figure 1F), and caecal atresia (Figure 1G). Expected (i.e., normal) changes in organ morphology during development notwithstanding, these abnormalities in general phenotype were represented at all gestational ages of *Fgf10* null embryos assessed. Further, as our experimental question and aim placed primary focus on abdominal foregut morphology, we did not undertake detailed comparisons of these general phenotypic features during *Fgf10* null embryo development, e.g., between tm1 and tm2.

The general phenotype of tm1 and tm2 *Fgf10* heterozygotes mirrored that of wild-type (i.e., normal) litter mates, including morphologically normal limb buds, bilateral and branched lungs, normal appearance of tracheal rings and no caecal atresia. Finally, whilst the morphology and ultrastructure of some organs in *Fgf10* heterozygous mice is known to be abnormal (Jaskoll et al., 2005; El Agha et al., 2012), these were not formally assessed for the tm1 and tm2 strains, with the exception of foregut morphology as detailed below.

### Duodenal Phenotype Characterization

**FIGURE 2:**
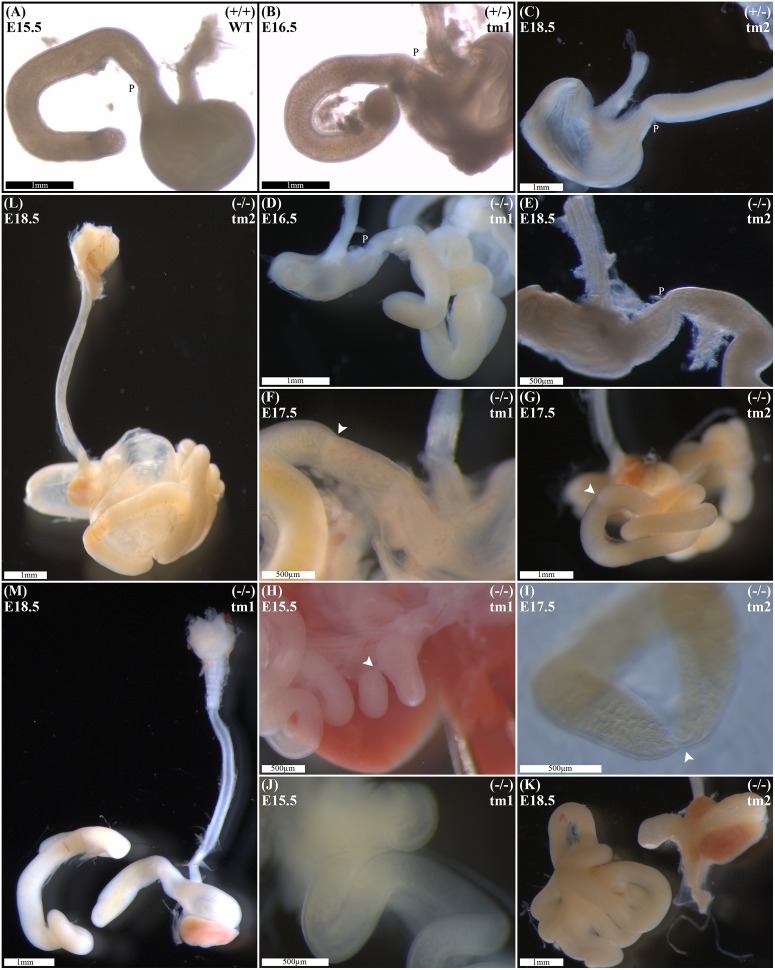
Duodenal phenotype characterization. Normal gastric, pyloric, and duodenal morphology was demonstrated by wild type embryos **(A)**, as well as *Fgf10* heterozygous embryos for tm1 **(B)**, and tm2 **(C)** strains. Null *Fgf10* embryos universally demonstrated microgastria, but duodenal morphology varied according to presence and type of DA. Null embryos provided examples of: normal continuity and morphology of the duodenum for tm1 **(D)** and tm2 **(E)**; type 1 DA, tm1 **(F)** and tm2 **(G)**; type 2 DA, tm1 **(H)** and tm2 **(I)**; and type 3 DA, tm1 **(J)** and tm2 **(K)**. **(L)** Type 2 DA demonstrating a “double bubble” with significantly dilated proximal duodenum; tm2. **(M)** Type 3 DA demonstrating an intact esophagus, in the presence of tracheal atresia; tm1. Annotations denote genotype, scale bars, and location of pylorus, P. Arrows indicate location atresia in type 1 DA and type 2 DA examples. Gestational ages for each panel are as stated.

Wild-type litter mates provided controls for normal gastric, pyloric, and duodenal morphology (Figure 2A). Without exception, *Fgf10* heterozygous embryos also demonstrated normal gastroduodenal morphology (Figures 2B,C), as distinct from *Fgf10* null embryos.

Of 61 *Fgf10* null embryos, 45 (74%) demonstrated DA. With DA observed in 21 (75%) of tm1, and 24 (73%) of tm2 *Fgf10* null embryos. *Fgf10* null embryos without DA did, however, demonstrate microgastria, which was a universal phenotypic feature for the *Fgf10* null cohort (Figures 2D,E). Both tm1 and tm2 strains provided examples for all three DA types: tm1 with 9 (43%) type 1, 4 (19%) type 2, and 8 (38%) type 3 DA; tm2 with 13 (54%) type 1, 4 (17%) type 2, and 7 (29%) type 3 DA (Figures 2F–K). The small sample sizes for each strain precluded meaningful comparison of relative type frequency between the strains and gestational ages.

Duodenal atresia in *Fgf10* null embryos reiterated the type morphology of DA in humans. In each case, the morphology of DA was recognizably distinct from the physiological luminal narrowing at the junction between the stomach and duodenum, i.e., pylorus (Figures 2A–E). Type 1 DA is characterized in these examples (Figures 2F,G) by continuity of the outer (serosal) aspect of the fetal gut wall, whilst the lumen is obstructed by a web at the site of atresia. Other examples of type 1 DA (not shown in Figure 2) demonstrate stenosis of the gut lumen, such that the lumen is notably and abnormally narrowed, whilst epithelial continuity persists. Type 2 DA (Figures 2H,I,M) represents a more severe duodenal anomaly, in which the gut wall and lumen are discontinuous, albeit a bridge or span of tissue maintains a physical connection between the two ends. Finally, type 3 DA (Figures 2J–L) is the most severe duodenal phenotype, recognized here by complete disconnection of the atretic gut ends.

### Other Intestinal Phenotype Characterization

Given the recognized association between DA and esophageal atresia in humans, DA-affected *Fgf10* null embryos were analyzed for esophageal continuity. Figure 2L (tm1) and Figure 2M (tm2) provide representative images of the universally intact esophagus for these embryos, i.e., no esophageal atresia. The 100% penetrance of caecal atresia in tm1 and tm2 *Fgf10* null embryos has been noted above.

## Discussion

### A Necessary Model

A limited understanding of the etiology of DA presently restricts the design and assessment of strategies to prevent or ameliorate the phenotype of DA in humans. As it is not possible to directly investigate the pathogenesis of DA in human embryos, a suitable animal model is required to advance knowledge toward such translation. Although the association between DA and Down syndrome is the best understood genetic link with DA in humans, the animal models for Down syndrome are not applicable here as they universally fail to demonstrate DA or indeed any other Trisomy 21-associated gastrointestinal abnormalities (Delabar et al., 2006).

Like Trisomy 21 in humans, interruption of FGF10/FGFR2b signaling is the best demonstrated genetic link to DA in mice (Fairbanks et al., 2004; Kanard et al., 2005; Botham et al., 2012; Reeder et al., 2012). Despite this, mice lacking expression of either *Fgf10* or its receptor gene did not provide a promising model for future study due to relatively poor penetration of the DA phenotype with DA present in only 35–45% (Fairbanks et al., 2004; Kanard et al., 2005; Botham et al., 2012; Reeder et al., 2012). Further, these strains failed to reiterate in mice the full spectrum and distribution of DA types seen in humans within a single strain with, at times, unexplained contradiction in the distribution of DA types demonstrated (Kanard et al., 2005; Reeder et al., 2012).

### An Improved and Promising Model

The DA penetrance and types evident in our novel CRISPR-derived, *Fgf10* knockout mouse strains, reported here as tm1 and tm2, represent an important and positive development in the field. In distinction to previous reports, our novel *Fgf10* null embryo strains demonstrate both a significantly higher penetrance (74%), as well as examples for all three morphological types of DA.

The basis for the differences in DA penetrance and DA type distribution between our and previously reported *Fgf10* knockout mouse strains remains unclear. The strains are of consistent background, namely C57/Bl6 (Fairbanks et al., 2004; Kanard et al., 2005), and whilst the genetics differ, each represents a nonsense mutation (Eppig et al., 2006). The exact genetics, and its interplay on downstream targets, may well be playing a role in penetration of the DA phenotype. Interestingly, Reeder et al. (2012) observed that the addition of haploinsufficiency of retinaldehyde dehydrogenase 2 (*Raldh2* ±) to *Fgfr2IIIb* homozygous null embryos resulted in a reduced penetrance of DA, accompanied by a less severe DA phenotype. Of note, type 2 DA was evident in *Fgfr2IIIb*(−/−); *Raldh2*(±) but not in *Fgfr2IIIb*(−/−) mouse embryos (Reeder et al., 2012). Reeder et al. (2012) results could be interpreted as demonstrating a relationship between DA penetrance and type distribution in the murine model. If this were so, a model demonstrating all DA types might be expectedly undermined by weak penetrance. However, our results demonstrate both improved penetrance as well as expression of all three DA types, affirming applicability of the tm1 and tm2 strains presented here as a promising model of DA.

Whilst the performance of the tm1 and tm2 strains is enhanced, neither strain demonstrated complete penetrance of DA. Incomplete penetrance of the duodenal phenotype is distinct from other phenotypic features of *Fgf10* null embryos, which demonstrate complete penetrance, e.g., absent limbs, absent lungs, and caecal atresia. Presently undefined developmental differences notwithstanding, we consider the incomplete penetrance of DA indicates redundancy in the requirements for “normal” duodenal morphogenesis. As such, tm1 and tm2 *Fgf10* null embryos without DA provide a rich population for future study, to discern signature signaling differences when compared with wild-type, heterozygote and DA-affected *Fgf10* null embryos. Understanding such differences may reveal key etiological components responsible for DA, as well as attributes for exploitation in future translations to achieve normal duodenal development in humans with a genetic predisposition to DA.

### A Hypothesis-Driven and Disciplined Model

Fundamentally, we hypothesize that the etiology of DA in humans is genetic, with the causative genetic changes located downstream of the FGF10/FGFR2b signaling pathway. This downstream locus may account for the incomplete penetrance of DA in the murine model as discussed previously. Further, interruption of downstream signaling may account for both the lack of either *FGF10* or *FGFR2b* gene deletion in human DA cases previously screened for this (Tatekawa et al., 2007), as well as the absence of non-survivable associations of *FGF10* deletion in humans with DA, such as pulmonary agenesis.

However, before we may begin to scrutinize and develop this hypothesis within our *Fgf10* knockout model, our rigor must be first directed to gaining a better understanding of the temporo-spatial expression patterns relevant to the Fgf10/Fgfr2b signaling pathway in the murine fore and midgut. Existing similar expression patterns have focused on the more anterior foregut (Nyeng et al., 2007, 2008), the question of gut boundary regionalization (as reviewed in San Roman and Shivdasani, 2011), or lacked sufficient temporo-spatial resolution to adequately inform further investigation within our DA model (Fon Tacer et al., 2010). To understand the expression pattern of *Fgf10* in the developing duodenum, we plan to exploit the fidelity and imaging possibilities afforded by the *Fgf10*^CreERT2^_tomato^flox/flox^ [B6-*Fgf10*<tm1.1(cre/ERT2)Sbel> Gt(ROSA)26Sor<tm9(CAG-tdTomato)Hze>] and *Fgf10*^flox/^^+_^tomato^flox/flox^ [B6.*Fgf10*<tm1.2 Sms>] mouse strains (El Agha et al., 2012). Using these powerful developmental biology tools, we will assess stage- and tissue-specific expression of *Fgf10*, and correlate this with coincident expression patterns for *Fgfr2b* and their collective downstream targets. Expression patterns will be assessed using immunohistochemistry, as well standard and quantitative PCR methods.

Having established normal expression patterns, we will then be able to compare and contrast expression of the same signaling pathway components in tm1 and tm2 *Fgf10* heterozygote and *Fgf10* null embryos, both those with and without DA. The use of staged-gestation embryos, including embryos from earlier gestation than the previous threshold of E15.5, will allow us to determine when and how in development molecular divergence between these genetically distinct mice arises. These temporo-spatial molecular characteristics will then be correlated with concurrent normal or abnormal morphogenic changes in the developing duodenum.

### A Testable and Translational Model

The ultimate aim of our DA research is to identify targets for translational therapies to prevent or ameliorate DA in humans. In humans, DA is associated with a wide range of associated anomalies, the presence of which negatively impact prognosis (Hemming and Rankin, 2007; Choudhry et al., 2009). We have established an ethics-approved (#DB077) clinical database for all children managed with DA at The Royal Children’s Hospital, Melbourne since 2000. Within this cohort of more than 100 DA patients (unpublished data), we have identified a thought-provoking subset with associated anomalies akin to Fgf10-Fgfr2b signaling-related defects, namely craniofacial, limb and lung anomalies (Sekine et al., 1999; De Moerlooze et al., 2000; Teshima et al., 2016). We consider this human subset to be at particular “risk” of an underlying genetic changes impacting the FGF10/FGFR2b signaling pathway, and plan to undertake detailed clinical genetic phenotyping and exon sequencing of phenotypically homogenous patient groups. Candidate mutations thus identified will be tested by introducing these variants into mice using CRISPR, and confirming whether this (or these) mutations do indeed generate DA in these increasingly bespoke murine models. Moreover, a putative genetic mutation (or mutations) responsible for DA would represent a landmark development in the understanding of normal gut morphogenesis and DA, opening the door to superior genetic and antenatal counseling, as well as potential future therapies for DA-affected fetuses and families.

## Author Contributions

WT conceived the project and prepared the study design, methodology, molecular biology techniques, performed the microsurgical tissue dissection, image capture, analyzed and interpreted the results, and wrote and finalized the manuscript. MJ assisted in study design, molecular biology techniques, microsurgical tissue dissection, image capture, results analysis and interpretation, preparation, and finalization of the manuscript. LH assisted in design and performance of mouse strain derivation using CRISPR technique. IS assisted in design and performance of mouse strain derivation using CRISPR technique and finalization of the manuscript. AC assisted in design and performance of molecular biology techniques. GS performed the molecular biology techniques. SK assisted in study design, results interpretation, and finalization of the manuscript. RL assisted in study design, establishment of methodology, molecular biology techniques, and finalization of the manuscript. JH mentored WT, assisted in study design, results interpretation, and finalization of the manuscript.

## Conflict of Interest Statement

The authors declare that the research was conducted in the absence of any commercial or financial relationships that could be construed as a potential conflict of interest.
