# Supplementary material for: FGF10 and the Mystery of Duodenal Atresia in Humans
Source: Front Genet. 2018 Nov 9;9:530. doi: 10.3389/fgene.2018.00530 (PMC6238159; doi:10.3389/fgene.2018.00530)
Supplement: Supplementary file 1 [file Table_1.DOCX]

Supplementary Material

**FGF10 and the mystery of
duodenal atresia in humans**

**Authors**

Warwick J. Teague**^1,2,3,4^*, Matthew L.M. Jones*^1,3,4^*, Leanne Hawkey^5^, Ian M. Smyth*^5,6,7^*, Angelique Catubig*^1^*, Sebastian K. King*^1,2,4,8^*, Gulcan Sarila*^1^*, Ruili Li*^1^*, John M. Hutson*^1,2,9^*

**Affiliations**

*^1^ F. Douglas Stephens Research Laboratory, Surgical Research Group, Murdoch Children’s Research Institute, Melbourne, VIC, Australia*

*^2^ Department of Paediatrics, University of Melbourne, Melbourne, VIC, Australia*

*^3^ Discipline of Surgery, Sydney Medical School, University of Sydney, Sydney, NSW, Australia*

*^4^ Department of Paediatric Surgery, The Royal Children’s Hospital, Melbourne, VIC, Australia*

*^5^ Australian Phenomics Network, Department of Anatomy and Developmental Biology, Monash University, Melbourne, VIC, Australia.*

*^6^ Department of Anatomy and Developmental Biology, Monash Biomedicine Discovery Institute, Monash University, Melbourne, VIC, Australia.*

*^7^ Department of Biochemistry and Molecular Biology, Monash Biomedicine Discovery Institute, Monash University, Clayton, Melbourne, VIC, Australia*

*^8^ Department of Gastroenterology & Clinical Nutrition, The Royal Children’s Hospital, Melbourne, VIC, Australia*

*^9^ Department of Urology, The Royal Children’s Hospital, Melbourne, VIC, Australia*

**Correspondence**

Associate Professor Warwick Teague

warwick.teague@rch.org.au

# Supplementary Materials and Methods

## DNA extraction

For genotyping, tail or ear clippings were placed in 600µl of extraction buffer (50mM of NaOH, pH 8.5), centrifuged at 14,000rpm for 1min and placed on a heat block for 15mins at 100˚C. Each sample was vortexed for 1min, then returned to the heat block for a further 5mins at 100˚C. Samples were removed from the heat and allowed to cool for 20mins at room temperature. Genomic DNA was precipitated with 50µl of 1M Tris-HCl (pH 8.0), mixed well in readiness for PCR. DNA concentration was measured using Nanodrop spectrophotometer (Thermo Scientifics, 2000) at 260nm wavelength.

## Primer targets and PCR screening strategy for tm1 mice

The primer sequences used to genotype tm1 mice are as shown in Table 1.2.1. These primers were then used for the PCR program which was conducted on genomic DNA as per Table 1.2.2.

###### **Table 1.2.1 The primer sequences used to genotype tm1 mice**

| Primer ID | Primer sequence 5’ – 3’ | PCR target and size | |
| --- | --- | --- | --- |
|  |  | **Wild Type** | **Mutant** |
| Forward | GGAGTGTAGATCATTACATGGC | 348bp | 215bp |
| Reverse | GTGAGGATACCATCTCTTTCTGTCC |  |  |

Primer set for establishing the PCR for the detection of tm1 mutation

**Table 1.2.2 The optimized PCR conditions for identifying tm1 deletion on exon 1 of *Fgf10***

| Reaction Mix | Reaction Conditions | | |
| --- | --- | --- | --- |
| Genomic DNA | 1$\mu$L | *Initial denaturation* | 95 ˚C for 3 mins |
| Primers | 0.05mcM each | *Denaturation* | 95 ˚C for 30 s |
| GoTaq (Promega Cat:M3001)  5x GoTaq Buffer | 0.025U  1.5mM | *Annealing* | 60 ˚C for 30 s |
| dNTP  MgCl | 0.2mM  1.5mM | *Extension* | 72 ˚C for 1 min  *(return to denaturation for 36 cycles)* |
| H20 to make a reaction volume of | 20$\mu$l | *Completion* | 72˚C for 5 mins |

Polymerase chain reaction (PCR), Minutes (mins), Seconds (s)

## Primer targets and PCR screening strategy for tm2 mice

The primer sequences used to genotype tm2 mice are as shown in Table 1.3.1. These primers were then used for the PCR program which was conducted on genomic DNA as per Table 1.3.2. On completion of the PCR, a Nsi1 digestion protocol was conducted as per Table 1.3.3 to split the wild-type allele to 242bp and 148bp.

###### **Table 1.3.1 The primer sequences used to genotype tm2 mice**

| Primer ID | Primer sequence 5’ – 3’ | PCR target and size | |  |
| --- | --- | --- | --- | --- |
|  |  | **Wild Type** | **Mutant** | |
| Forward | GGAGTGTAGATCATTACATGGC | 390bp  (242bp and 148 bp after Nsi1 digestion) | 377bp | |
| Reverse | GAATTCAGGGCTATGTCTTTGC |  |  |  |

Primer set for establishing the PCR for the detection of tm1 mutation

**Table 1.3.2 The optimized PCR conditions for identifying tm2 deletion on exon 1 of *Fgf10***

| Reaction Mix | Reaction Conditions | | |
| --- | --- | --- | --- |
| Genomic DNA | 1$\mu$L | *Initial denaturation* | 95 ˚C for 3 mins |
| Primers | 0.1mcM each | *Denaturation* | 95 ˚C for 30 s |
| GoTaq (Promega Cat:M3001)  5x GoTaq Buffer | 0.05U  3mM | *Annealing* | 60 ˚C for 1 min |
| dNTP  MgCl | 0.4mM  3mM | *Extension* | 72 ˚C for 1 min  *(return to denaturation for 30 cycles)* |
| H20 to make a reaction volume of | 10$\mu$l | *Completion* | 72˚C for 5 mins |

Polymerase chain reaction (PCR), Minutes (mins), Seconds (s)

**Table 1.3.3 Nsi1 digestion protocol for the Wild-type allele**

| Reaction Mix | Reaction Conditions | | |
| --- | --- | --- | --- |
| PCR products | 10$\mu$L |  |  |
| Nsi1 (Promega, Cat:R5351) | 0.05U | *Digestion* | 37 °C for 90mins |
| 10x Nsi buffer  10x BSA | 1$\mu$L  1$\mu$L |  |  |
| H20 to make a reaction volume of | 20$\mu$l |  |  |

Polymerase chain reaction (PCR), Minutes (mins), Seconds (s)

## Gel electrophoresis

After PCR amplification, 1.5% agarose gel electrophoresis was performed in 1x Tris-Boric acid-EDTA (TBE) buffer (90mM Tris-boric, 2mM EDTA) at 100V for 35mins, using 1kb DNA ladder (New England Biolabs, Cat: N3200L). PCR products were stained with Gel Red (Gene Target Solutions, Cat:41003) and visualized with Bio-Rad Gel Doc at standard UV transilluminator 302nm (Life Sciences, 2001).

## RNA Extraction

Tissue samples isolated from tm1 and tm2 strains were minced and rapidly homogenized in 500µl of lysis buffer (Qiagen, Cat:74104) using homogenizer. Samples were then centrifuged at max speed for 3mins and RNA solution was transferred to RNasey® Mini Kit spin columns (Qiagen, Cat:74104), span down at 12000rpm for 30 seconds discarding the flow through each time. Columns were washed with RW1 buffer (from Qiagen mini RNasey Kit) centrifuged once again for 30 seconds at 12000rpm facilitating biding to the spin columns. A mixture of DNaseI (Qiagen, Cat:79254) and RDD buffer was made (1:7 dilution, RDD buffer provided in Kit) and placed onto the spin columns containing the bound RNA for 15 minutes at room temperature. Once again, RW1 buffer was added to the spin columns and span down at 12000rpm for 30 seconds, followed by two washes of RPE buffer with the same conditions. Spin columns were dry span at max speed for 2 minutes with a new collection tube to remove any residue of wash buffer on the column membrane. The RNasey spin column was air dried for 10 minutes at room temperature. Twenty to thirty microliters of elution buffer (nuclear free H_2_O) were added to the spin columns incubated for 1 minute and span down into a new 1.5ml tube at 12000rpm for 1min. This extracted the RNA from the spin column into the new tube then stored at
-80°C until required for analysis.

**1.5.1 Determining the concentration and quality of RNA**

RNA concentration was measure using Nanodrop spectrophotometer (Thermo Scientifics, 2000) at 260nm wavelength. Two microliters of sample were placed on the spectrophotometer reader and measured, concentration was determined and nuclease free water was used as a blank. To evaluate the quality of the RNA sample, the absorbance at 260nm were compared to the wavelengths of 280nm and 230nm in order to determine the organic and protein contamination within the RNA samples. The samples that read 260/290 and 260/230 ratios close to 2 were accepted as a good quality of RNA.

**1.5.2 Complementary DNA synthesis and**

Complementary DNA (cDNA) was synthesized from the isolated RNA using BioRad iScript Advanced cDNA kit (BioRad, Cat: 170-8842). The synthesis was performed in a thermal cycler with the following conditions; 25˚C for 10mins, followed by 37˚C incubation for 2 hours, then at 85˚C for 5mins to stop the enzymatic reaction and then 4˚C indefinitely. The total volume of cDNA with nucleus free water was adjusted to 10ng/μl and stored at -20˚C. 2μl of the cDNA was used as a template for real time quantitative polymerase chain reaction (RT-qPCR).

**1.5.3 Real-time quantitative Polymerase Chain Reaction (RT-qPCR)**

Nucleotide sequences for *Fgf10* mRNA (*Fw* 5’-CACCTATGCATCTTTTAACTGGC-3’) (*Rv* 5’- TCTATGTTTGGATCGTCATGGGG-3’) was used and expression was normalized to *Rpl32* (*Fw* 5’-GAGGACCAAGAAGTTCATCAGG-3’) (*Rv* 5’-CATTGTGGACCAGGAACTTGC-3’) were ordered from Sigma-Aldrich and resuspended in TE buffer (100mM Tris, 1mM EDTA) to make at 100pmol/μl. For qPCR reaction, the primer stock was diluted 1:10 for 10pmol/μl in reaction mixture. cDNA samples were added onto each well of a 96 well optical plate (Applied Biosystems, Cat: 4346906) and measured by GoTaq qPCR (Promega, Cat:A6001) for real time quantitative polymerase chain reaction (RT-qPCR). The results were analyzed using the 7500 SDS software (Applied Biosystems) and relative expression was calculated as (Gene Ct –*Actb* Ct =CΔt). To determine the fold change, the formula was first calculated (ΔΔCt=Untreated CΔt –Treated CΔt), then as Fold Change=2^-(ΔΔCt). Statistical analysis was performed in Prism 7.0 (GraphPad Software). Error bars on the results are presented as a standard error of the mean (SEM). Statistical significance was evaluated using Student’s unpaired t-test; p-value of <0.05 was considered significant.
